# Degradation Behavior In Vitro of Carbon Nanotubes (CNTs)/Poly(lactic acid) (PLA) Composite Suture

**DOI:** 10.3390/polym11061015

**Published:** 2019-06-08

**Authors:** Shuqiang Liu, Gaihong Wu, Xiaogang Chen, Xiaofang Zhang, Juanjuan Yu, Mingfang Liu, Yao Zhang, Peng Wang

**Affiliations:** 1 College of Textile Engineering, Taiyuan University of Technology, Taiyuan 030024, China; liushuqiang8866@126.com (S.L.); xiaofangfang521a@163.com (X.Z.); yuxiaoyuy@126.com (J.Y.); lmf118119@163.com (M.L.); zy32164872@126.com (Y.Z.); tyut19834520797@163.com (P.W.); 2 School of Materials, The University of Manchester, Manchester M13 9PL, UK

**Keywords:** poly(lactic acid), carbon nanotubes, degradation behavior, composite suture

## Abstract

Poly(lactic acid) (PLA) suture can be absorbed by the human body, and so have wide applications in modern surgery operations. The degradation period of PLA suture is expected to meet with the healing time of different types of wounds. In order to control the degradation period of the PLA suture, the carbon nanotubes (CNTs) were composited with PLA suture, and the degradation experiment in vitro was performed on sutures. The structure and properties of sutures during degradation, such as surface morphology, breaking strength, elongation, mass and chemical structure, were tracked and analyzed. The results indicated that the degradation brought about surface defects and resulted in 13.5 weeks for the strength valid time of the original PLA suture. By contrast, the strength valid time of the CNTs/PLA suture was increased to 26.6 weeks. Whilst the toughness of both the pure PLA and CNTs/PLA sutures decreased rapidly and almost disappeared after 3 to 4 weeks of degradation. The mass loss demonstrated that the time required for complete degradation of the two sutures was obviously different, the pure PLA suture 49 weeks, while CNTs/PLA sutures 63 to 73 weeks. The research proved that CNTs delayed PLA degradation and prolonged its strength valid time in degradation.

## 1. Introduction

Medical sutures are made from Poly(lactic acid) (PLA) which is derived from agriculture products. It has several attractive features, such as harmless, biocompatibility, biodegradability, and high strength [1,2,3]. Such a suture can degrade into CO_2_ and H_2_O during wound healing, which will avoid the pain caused by the unnecessary suture removal [4,5,6]. For this reason, PLA sutures achieved a wide application in modern surgery operations, especially operations for internal organs and some special sites, such as face and neck, circumcision, colostomy [7,8,9].

Different types of wounds heal with different paces as indicated in Table 1 [10,11], and PLA sutures are expected to provide with correspondent degradation time. Being unable to control the degradation time would lead to ineffectiveness of PLA sutures in wound management [12,13,14]. It is essential to design the PLA sutures with controlled degradation time, and the core issue of this is to regulate the degradation rate of PLA sutures.

So far, many methods have been adopted to regulate the degradation periods of PLA sutures. Firstly, the simple way to control the degradation time of suture was achieved by adjusting the suture diameter, for thicker sutures would generally exhibit longer degradation time [15]. However, thinner sutures always have low strength to fasten the wound, and they could cut into the wound in a wound closure. In comparison, thicker sutures would usually cause big perforations and thus deteriorate the wound further. As a result, the range of appropriate diameters of sutures was limited. In the case of multifilament sutures, the size of the filament fibers would affect the degradation performance too, with finer fibers degrading faster. In addition, the degradation time and rate of PLA sutures could be regulated by copolymerization or blend with some high molecular materials such as polyethylene glycol (PEG) and polyglycolic acid (PGA). Toncheva et al. [16] copolymerized PEG with PLA and obtained high molecular weight segmented copolymer. Their results indicated that the addition of PEG segment into PLA molecular chain effectively enhances the PLA flexibility and shortens the degradation time compared to pure PLA. Blending easily degradable biomaterials, such as PGA and PEG, with PLA has also been proven to accelerate the degradation rate of PLA composite, but the strength of composite is usually decreased [17,18,19]. Thirdly, some nano-scale inorganic materials, e.g., nano-SiO_2_ [20] and nano-montmorillonite (nano-MMT), blended with PLA, have also been used to regulate the degradation rate and time of PLA-based sutures. Min et al. [21] mixed nano-MMT with PLA and produced PLA/nano-MMT fibers from this mixture, and the fiber demonstrated degradation acceleration compared to the pure PLA fiber, but the mechanical properties of the new fiber was found to decrease. Fourthly, the degradation rate of the PLA fibers can also be regulated by changing their surface characteristics. Benyathiar et al. [22] applied gamma and electron beam irradiation to erode the PLA fiber surface for enhanced hydrolysis and biodegradability. Zhang et al. [23] grafted polyvinylpyrrolidone (PVP) onto PLA molecular chain on the surface of the fiber aiming to increase the water absorption of PLA fiber, thus enhancing the degradation of PLA fiber. This method, similar to previous ones, would also reduce the mechanical properties of the PLA fiber system.

It is obvious that the techniques reviewed above regulating the fiber degradation mainly in the direction of shortening the degradation time. In practice, however, materials with prolonged degradation time are also required for closure of different types of wounds, which heal with a different length of time, as indicated in Table 1. It is equally important to slow down the degradation rate of PLA fibers as well as to speed up as reinforcement and degradation are just two opposite sides for fibers.

It can be seen from the literature that the lower the strength of the suture, the faster the degradation rate and the shorter the degradation time, and vice versa. Hence, for PLA based fibers, increasing the fiber strength would prolong the degradation time and slow down the degradation rate. There are many ways to increase the strength of PLA based sutures, and adding nano-reinforcing materials such as carbon nanotubes (CNTs) and graphene into the PLA fiber matrix has been reported to be an effective and popular method [24,25,26]. The carbon nanotubes (CNT_S_) are linear nanomaterials with ultra-high strength, excellent electrical conductivity and other virtues. Especially, the CNTs are non-toxic and harmless to the human body, and the degradation products can be excreted by human body functional tissues. The CNTs can be applied to make artificial bone, tendon, scaffold, and other biomedical tissues [27,28,29]. This research proposes the addition of carbon nanotubes (CNTs) into the PLA matrix to increase the strength of suture and thus to achieve the prolonged degradation time. Together with the achievements reported by previous researchers, this work will enable the sutures to have controlled degradation rate and time.

In this research, the CNTs/PLA sutures with different contents of CNTs were prepared. These sutures were put into in vitro degradation fluid for the evaluation of degradation behavior of the sutures. Scanning electron microscopy (SEM) and other characterization equipment were employed to observe the degradation process of the CNTs/PLA sutures, during which the suture strength, mass loss, and appearance were measured and evaluated. Through this investigation on the influence of CNTs contents on CNTs/PLA suture degradation, this paper attempts to establish an effective way to retard the degradation of PLA based suture and hence to achieve control of the suture degradation rate and time.

## 2. Materials and Methods

### 2.1. Materials

The PLA resin slices (6202D) used in this research were produced by Nature Works Co. Ltd., Blair, NE, USA, with number-average molecular weight (*M*_n_) being 51,000 g/mol. The multi-walled CNTs were obtained from Chengdu Organic Chemistry co., Ltd. (Chengdu, China), with 10–20 nm in diameter, 10–30 μm in length and with 3.06 wt% hydroxyl content. The silane coupling agent (KH570), CH_2_=C(CH_3_)COOC_3_H_6_Si(OCH_3_)_3_, used in the research was produced by Nanjing Capatue Chemical Co. Ltd., Nanjing, China.

### 2.2. Preparation of CNTs/PLA Sutures

Four steps were followed to prepare the CNTs/PLA sutures, which were CNTs modification, preparation of CNTs/PLA master batches, melt spinning of PLA composite filaments, and then the compound process for twist, heat setting, and sterilization.

#### 2.2.1. CNTs Modification

Owing to the high surface energy, the CNTs needed be treated before being used to improve the compatibility and dispersity in the PLA matrix. Silane coupling agent (KH570) was used to modify CNTs.

Firstly, KH570 was diluted by water and methanol (water: methanol: KH570 = 8: 72: 20) and hydrochloric acid was added to regulate pH value to 3–4. After hydrolysis for 30 min at the room temperature, KH570 hydrolysis solution was achieved. There was 1.5 g of this solution and 10 g of CNTs put into a flask and was magnetically stirred for 5 h at 60 °C. The blend solution was then dried in a vacuum oven at 120 °C for 10 h [30,31,32]. The obtained residue was the modified CNTs (KH570/CNTs). The residue was ground into powder and kept in vacuum bottles. The dispersion of such modified CNTs was found to be greatly improved, as reported in previous publications [33,34].

#### 2.2.2. Preparation of CNTs/PLA Master Batches

To make the CNTs further disperse homogeneously in the PLA matrix, CNTs/PLA master batches were prepared before melt spinning.

**Figure 1:**
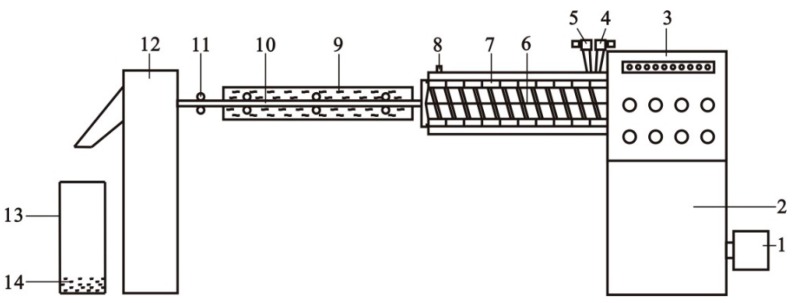
Diagrammatic sketch of the preparing machine for making carbon nanotubes (CNTs)/ Poly(lactic acid) (PLA) master batches. 1—main motor, 2—transmission case, 3—parameter adjustment controller, 4—main feed throat, 5—side feed throat, 6—twin screws, 7—ten heating zones, 8—vacuum pump, 9—cooling water bath, 10—CNT/PLA composite ribbon, 11—pulling rollers, 12—dicing cutter, 13—storage vat, and 14—CNTs/PLA master batches.

PLA resin slices were firstly dried by two steps in a Vacuum Drum Dryer (SZG-50, Changzhou Fuyi Drying Equipment Co. Ltd., Changzhou, China), i.e., 80 °C for 4 h and 100 °C for 8 h, with degree of vacuum −0.085 MPa ± 0.002 MPa. The dried PLA slices melt-blended with KH570/CNTs in a Twin-screw Extruder (CTE35, Nanjing Beikelong Apparatus Co. Ltd., Nanjing, China), as sketched in Figure 1. Then, after cooling and dicing, the CNTs/PLA master batches were prepared. The content of CNTs was 5 wt% of the total mass of the master batches, and the temperature of the ten heating zones of screw extruder was set as follows: Zones 1–2, 166–169 °C; Zones 3–4, 170–172 °C; Zones 5–6, 174–177 °C; Zones 7–8, 178–182 °C; Zones 9–10, 185–190 °C.

#### 2.2.3. Melt Spinning of CNTs/PLA Composite Filaments

**Figure 2:**
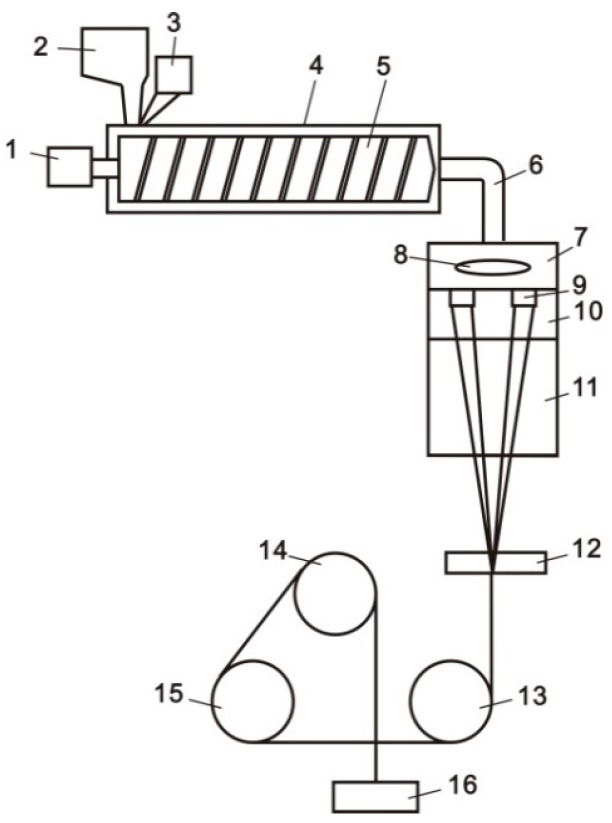
Sketch diagram of melt spinning of CNTs/PLA composite filaments. 1—screw machine, 2—main feed throat, 3—side feed throat, 4—four heating zones, 5—single screw, 6—elbow pipe, 7—spinning box, 8—metering pump, 9—spinning pack, 10—annealing device, 11—cooling device, 12—oil roller, 13—the first hot roller, 14—the second hot roller, 15—the third hot roller, and 16—winding device.

The pristine PLA slice and CNTs/PLA master batches were dried in vacuum at 120 °C for 12 h with degree of vacuum −0.083 MPa ± 0.002 MPa. The dried PLA master batches and slices were mixed in different proportions and melt spun into composite filaments by a Pilot-scale Fiber Spinning Machine (LHFJ030, Lanhua Textile Apparatus Co. Ltd., Wuxi, China). This process was sketched in Figure 2. The spinning technical parameters were listed in Table 2. The specification of obtained CNTs/PLA filaments was 150D/72F. It must be noted that the CNT content in PLA filament was selected as 0.5%, 1% and 2%. This is because that when the content of CNT in PLA fiber exceeded 2%, the spun filament was very easy to break or even failed to spin. Therefore, the content of CNT in PLA fiber cannot exceed 2%. In addition, the CNT content accuracy controlled by the spinning equipment was at least 0.5%. Therefore, in order to analyze the influence of CNT content on PLA fiber, the range of CNT content should be selected from 0.5% to 2%. The content of CNT was divided into high (2%), medium (1%) and low (0.5%) levels. In addition, the effects of three levels of CNT content on PLA fiber were studied.

#### 2.2.4. Twisting, Heating Setting, Sterilizing of CNTs/PLA Filaments

For medical suture applications, CNTs/PLA filaments will have to go through the process of twisting, heating setting and sterilizing. Firstly, the single-strand PLA filaments were dipped into ethyl alcohol under ultrasonic treatment at 20 °C and 40 KHz, in order to clean off the oil agents on the surface of the filaments. A Doubling Twisting Machine (DSTw-01, Jiacheng Electromechanical Equipment Co. Ltd., Tianjin, China) was used to twist these filaments with a twist factor of 205. The twisted yarns were heat set in a Vacuum Oven at 40 °C for 12 h. Then the yarns were further sterilized by dipping into medicinal alcohol for 10 min. After being dried in the oven at 40 °C for 20 min, the single-strand CNTs/PLA suture was prepared.

### 2.3. In vitro Degradation of CNTs/PLA Sutures

To investigate the degradation behavior of CNTs/PLA suture in human body, HANKs fluid was created to simulated the body fluid, which was composed of 8.0 g/L NaCl, 0.4 g/L KCl, 0.14 g/L CaCl, 0.1 g/L MgCl_2_, 0.06 g/L MgSO_4_, 0.06 g/L KH_2_PO_4_, 0.06 g/L Na_2_HPO_4_, 0.35 g/L NaHCO_3_, 1.0 g/L C_6_H_12_O_6_ [35,36]. The CNTs/PLA sutures were put into HANKs fluid which was maintained at 37 °C, the same as the temperature of inner body. The HANKs fluid was changed every two days, simulating the human circulatory system. At different time points in the degradation process, these sutures were taken out and rinsed with deionized water 4 times to remove the chemical residues. After that these sutures were dried in a Vacuum Oven at 35 °C for 6 h, and tested for their structures and mechanical properties.

### 2.4. Differential Scanning Calorimetry (DSC)

Suture crystallization behavior was analyzed by a differential scanning calorimeter (DSC6000, PerkinElmer, Fremont, CA, USA). Under nitrogen protection, the temperature was heated to 190 °C at 10 °C/min, then retained heat for 3 min, and following cooled to 30 °C at 5 °C/min.

### 2.5. Scanning Electron Microscope (SEM)

SEM investigations were performed on a JSM-6510LA (JEOL, Akashima, Japan) at 7 kV accelerating voltage to evaluate the fiber morphologies and fractured surfaces of sutures. All the samples were mounted on aluminum holders and coated with a thin layer of gold prior to observation.

### 2.6. Fourier Transform Infrared Spectroscopy (FT-IR)

The infrared spectra were obtained via FT-IR (TENSOR27, Bruker Optics, Karlsruhe, Germany) with a resolution of 4 cm^−1^ that scanned 50 times from 450 to 4000 cm^−1^ at the room temperature. All samples were taken using the conventional KBr disk method.

### 2.7. Mechanical Properties of CNTs/PLA Sutures

The tensile strength and elongation of CNTs/PLA sutures were tested by an Electronic Single Yarn Strength Tester (YG061FQ, Laizhou electronic instruments Co. Ltd., Laizhou, China) with the clamping length of 250 mm.

### 2.8. Mass Loss of CNTs/PLA Sutures

Before degradation test, the mass of PLA sutures (*M*_0_) were weighted by a Precision Electronic Balance. During degradation, the sutures were taken off at different time points and dried in a Vacuum Oven at 40 °C for 2 h, thus mass (*M*_1_) was further weighted. The mass loss of PLA sutures (*W*) was calculated by the Equation (1).

(1) $$W=\frac{M_{0}-M_{1}}{M_{0}}\times 100\%$$

## 3. Results and Discussion

### 3.1. The Crystallization Structure of CNTs/PLA Suture

**Figure 3:**
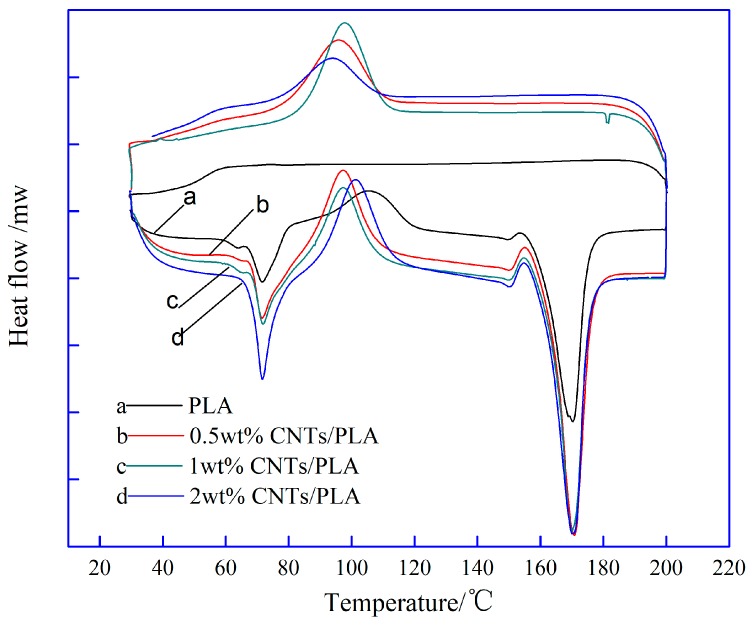
DSC curves of PLA sutures with different contents of CNTs.

The DSC curves of PLA suture and CNTs/PLA sutures were shown in Figure 3.

**Figure 4:**
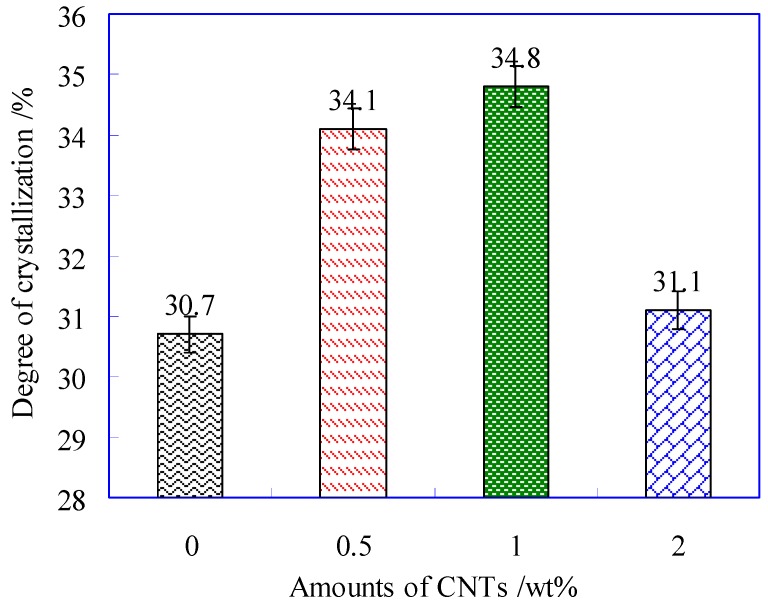
Degree of crystallization of PLA fiber with different contents of CNTs.

The crystallinity of sutures can be calculated by the Equation (2). Where Δ*H_m_* represents melting enthalpy of PLA (J/g); Δ*H_c_*, cold crystallization enthalpy of PLA (J/g); *λ*, mass fraction of PLA in composite suture; Δ*H_m,o_*, melting enthalpy of 100% crystal, its value was 93.6 J/g. Then the crystallinity of PLA suture with different contents of CNTs was obtained as shown in Figure 4.

(2) $$\chi_{c}\left(\%\right)=\frac{\Delta H_{m}-\Delta H_{c}}{\lambda \Delta H_{m}{}_{,o}}$$

It can be observed from Figure 4 that the crystallization firstly increased with the contents of CNTs, and then decreased. When at 1 wt% CNTs, the suture had the highest crystallinity. This is because that small amount of CNTs playing as a nucleating agent can induce crystallization, which accelerated the growth of crystal nucleus, and further improved the crystallinity of PLA suture. However, excessive CNTs (e.g., 2 wt%) aggregated identically, and the agglomeration would retard the growth of crystal nucleus, therefore the ability of inducing crystallization would decline.

### 3.2. The Surface Morphology of CNTs/PLA Sutures during Degradation

**Figure 5:**
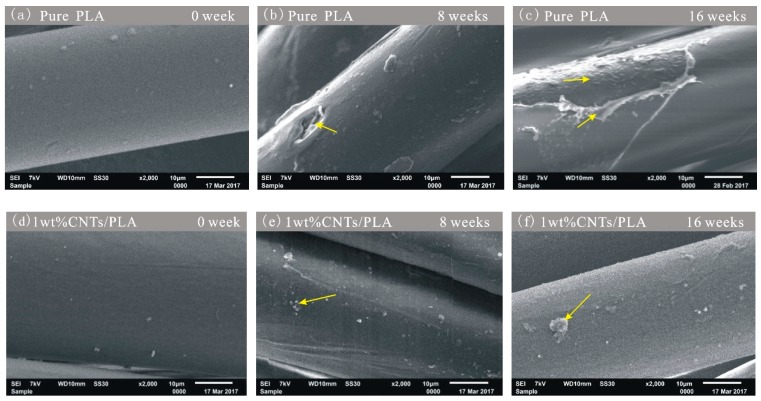
SEM images of surface morphology of sutures at different degradation time points: (**a**) pure PLA suture degraded for 0 week, (**b**) pure PLA suture degraded for 8 weeks, (**c**) pure PLA suture degraded for 16 weeks; (**d**) 1 wt% CNTs/PLA suture degraded for 0 week, (**e**) 1 wt% CNTs/PLA suture degraded for 8 weeks, (**f**) 1 wt% CNTs/PLA suture degraded for 16 weeks.

The degradation experiments in vitro were performed on pure PLA sutures and CNTs/PLA sutures. The surface morphology of sutures was measured by SEM, as shown in Figure 5.

The degradation SEM images of pure PLA sutures show that the surface of pure PLA suture (Figure 5a) is very smooth. With the further degradation, the surface of pure PLA sutures appeared many pits (Figure 5b) and some severe swellings and corrosions (Figure 5c). Thus, it indicates that the pure PLA fiber was gradually degraded from outside to inside. The degradation process of fiber the surface was not uniform, because the areas of suture, where many small fractures or defects appeared, were more easily eroded and hydrolyzed by the simulated body fluid. The degradation in these weak areas of PLA fiber was obvious quicker than that in other’s areas. With the further degradation, these weak areas were expanding, and then became the very weak link for the suture to rupture.

The images of CNTs/PLA sutures (Figure 5d–f) show that the surface of CNTs/PLA sutures became rougher and rougher with the further degradation, and appeared a number of small patches. Further, at the same degradation time points, the degradation degree of pure PLA sutures (i.e., Figure 5b,c) was much more serious than that of CNTs/PLA sutures (i.e., Figure 5e,f). It was because the CNTs reinforced PLA sutures and enhanced their crystallinity and orientation [34,37], thence the CNTs/PLA sutures degraded more slowly. These results indicate that CNTs can slow down the degradation rate of sutures, which will be further corroborated later.

**Figure 6:**
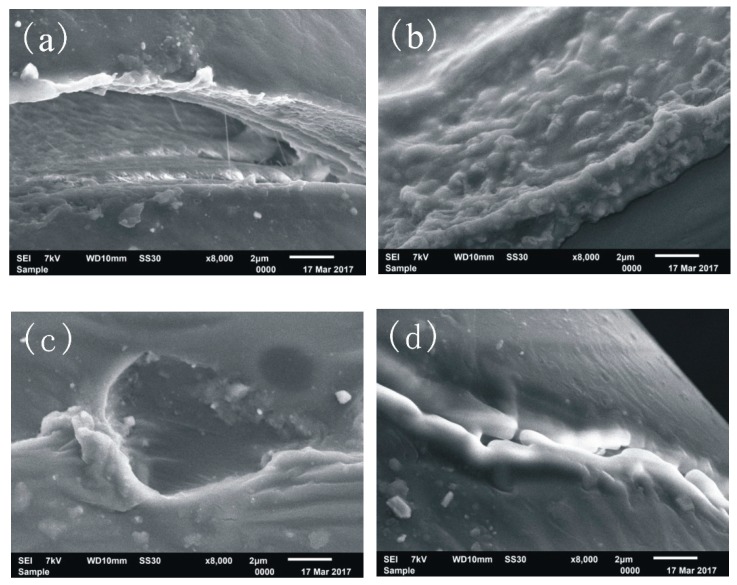
Four structure defects of sutures: (**a**) peeling, (**b**) swelling, (**c**) corrosion hole, (**d**) ditch.

During the process of degradation, the surface of suture would be degraded and formed some structure defects, as listed in Figure 6.

Figure 6a shows the defect of peeling, which was caused by the gradual degradation of the inner part of the suture, and the hard-degradable epidermis appeared, as a state of peeling. Figure 6b shows the defect of swelling. Since the simulated body liquid could permeate into the interior of suture, and then part of suture, consisting of the simulated body liquid, PLA and some degradation products, would be swelled. Figure 6c shows the defect of corrosion hole. The materials in the irregular circular area of PLA-base suture were soft and biodegradable, and then they were degraded severely and eroded deeply as a corrosion hole. Figure 6d shows the defect of ditch. There were some thin flaws on the surface of the PLA-base suture. The degradation would begin through these defects. Under the destructive action of degradation fluid, these thin flaws were extended, and eventually developed as big ditches. In the process of degradation, different structure defects would appear interactively, and would form the weak links for the PLA-base suture to rupture.

### 3.3. The Breaking Strength of CNTs/PLA Sutures during Degradation

**Figure 7:**
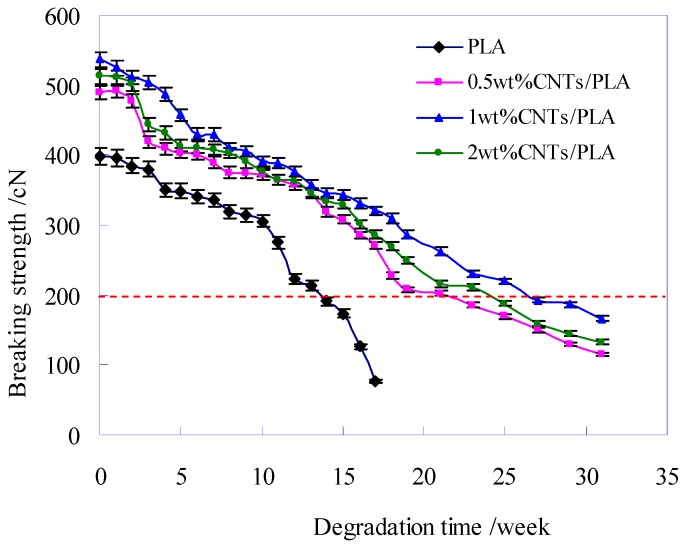
Breaking strength of pure PLA suture and CNTs/PLA sutures during degradation.

A specific wound needs certain time to be healed, the suture should maintain enough strength to prevent the wound cracking or separating [38,39]. Therefore, the mechanical property was a vital requirement for PLA-base suture in the process of degradation. The breaking strength of pure PLA suture and CNTs/PLA sutures during degradation were measured, as shown in Figure 7.

Before degradation, the initial breaking strength of PLA-base sutures was different and shown in Figure 7 at degradation time of 0. The initial breaking strength of CNTs/PLA sutures was much higher than that of PLA suture, especially that of the PLA-base suture with 1 wt% CNTs increased by 35% compared to pure PLA. Comparing the three CNTs/PLA sutures, it was found that the initial breaking strengths of PLA-base suture with too little (e.g., 0.5 wt%) or too much (e.g., 2 wt%) CNTs were not the highest, but the PLA-base suture with moderate amounts (e.g., 1 wt%) of CNTs had the highest initial breaking strength. This was because the CNTs possessed ultra-high strength and could improve the orientation, crystalline and homogeneity of PLA fiber matrix; hence the PLA-base suture could be effectively reinforced [40,41]. The initial breaking strength of PLA-base suture, added too little (e.g., 0.5 wt%) CNTs had more room to improve. On the other hand, too much (e.g., 2 wt%) CNTs would cause agglomeration in PLA-base suture, which could curb the increase of strength. Only added moderate and appropriate amount (e.g., 1 wt%) of CNTs, the CNTs/PLA suture had the highest strength.

Figure 7 also shows that the breaking strength of all sutures decreased with the degradation time, and the pure PLA suture decreased obviously faster than CNTs/PLA sutures, which suggested the addition of CNTs into PLA matrix prolonged the degradation time of suture, and slowed down the degradation rate. Based on the relationship of the degradation time and the breaking strength, the fitting regression equations were obtained, as listed in Table 3.

**Figure 8:**
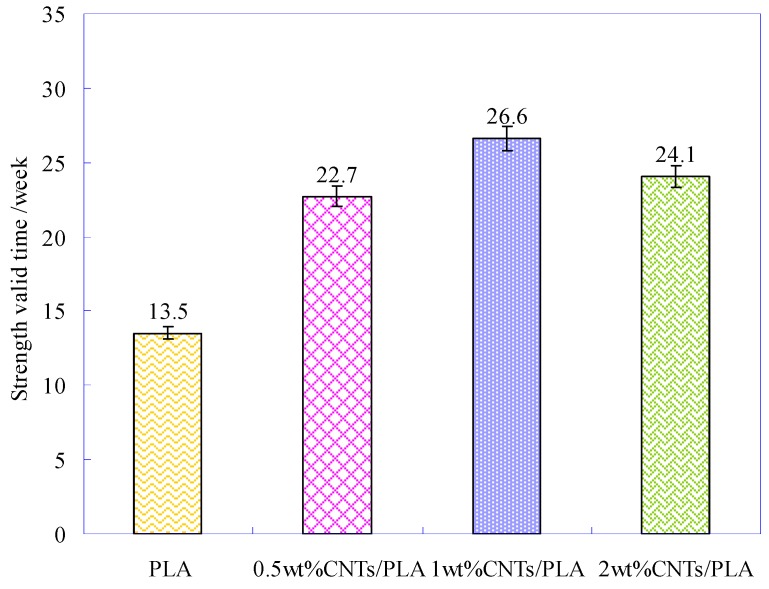
The strength valid time of pure PLA suture and CNTs/PLA sutures.

During degradation, the strength of the PLA-base suture would gradually decline. When the strength was not strong enough to support the expansion of the wound, the PLA-base suture would be broken, called as mechanical failure. The degradation time, corresponding to mechanical failure point of suture, is the valid time for PLA-base suture. In this research, the failure strength of PLA-base suture was about 200 cN (*y* = 200) (the red dotted line in Figure 5) [42,43,44]. According to the fitting equations, the valid times of different PLA sutures were obtained, as sketched in Figure 8.

Figure 8 shows that the strength valid time of pure PLA suture was 13.5 weeks, and the time for CNTs/PLA sutures was much longer. Especially for the PLA suture with 1 wt% CNTs, its valid time was 26.6 weeks, which was twice than that of pure PLA suture. Thus indicated that the CNTs could slow down the degradation and prolong the strength valid time of PLA-base suture.

### 3.4. The Breaking Elongation of CNTs/PLA Sutures during Degradation

**Figure 9:**
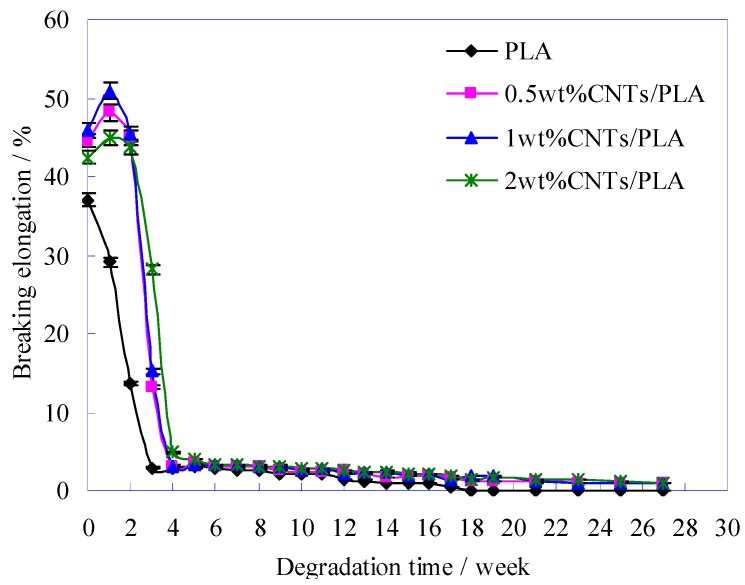
The variation of breaking elongation of PLA-base sutures with degradation time.

During the degradation process, the elongation of PLA-base sutures was also declining, as illustrated in Figure 9.

The curves change trends in Figure 9 shows that the initial elongations (at degradation time of 0) of CNTs/PLA sutures were higher than that of pure PLA suture, which indicated that the CNTs refined the crystalline grains and then improved the toughness of PLA suture efficiently [45]. With the degradation extension, the elongation of pure PLA suture declined obviously, and after 3 weeks its elongation had been very small (below 5%), that is to say, its toughness had almost disappeared. However, for CNTs/PLA sutures, during the first 1–2 weeks, their elongation increased instead. It might mainly because the absorbed water made CNTs and PLA macromolecules slide easily, so their elongation increased a little. However, with the time extension, more water entered in CNTs/PLA suture, the ester bonds of PLA macromolecules were destroyed and many structure defects emerged on surface of CNTs/PLA suture, then the elongation of CNTs/PLA suture decreased rapidly from the 3rd weeks, and at 4th week the elongation had declined to 5% and even to 0% in later degradation time.

When the PLA and CNTs/PLA sutures were used in surgical operations, their toughness should be paid more attention to prevent the hurt from the suture fracture due to the toughness losing.

### 3.5. The Mass Change of PLA Sutures during Degradation

In the process of degradation, the strength of PLA-base suture decreased gradually until the suture broke. After that, the suture has no positive effect, but the main part of PLA-base sutures was still in the human body, and then the remnants of the sutures would continue to be degraded and be absorbed by the body. The time of suture residue persisting in the body could affect the recovery of the wound. Therefore, after the suture broke, the residues of PLA-base suture were expected to be degraded and absorbed as soon as possible.

**Figure 10:**
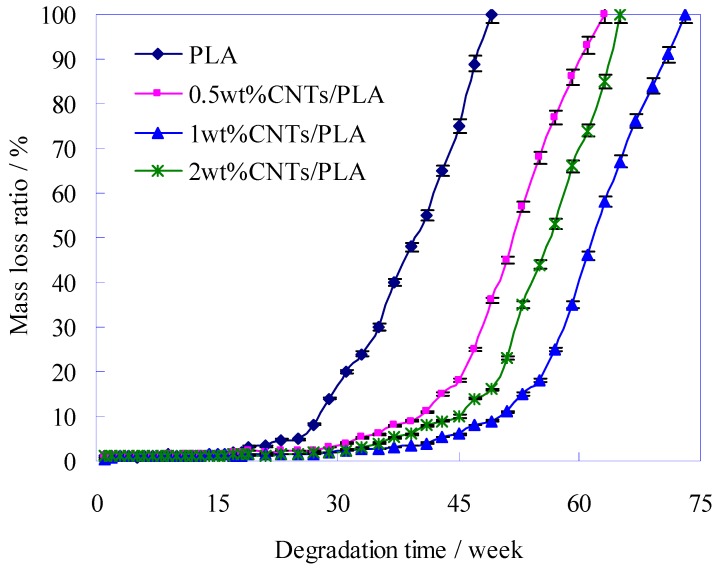
Change of mass loss ratio during degradation weeks.

The mass loss of PLA-base suture could be applied to evaluate the degradation and absorption rate of suture residue in the body. The mass loss of PLA-base sutures during degradation is shown in Figure 10.

It could be seen from Figure 10 that at an early stage, the ratio of degradation was very slowly before the 25th week for the pure PLA suture, before 35th week for the 0.5 wt% and 2 wt% of CNTs/PLA sutures, and before 45th week for the 1 wt% CNTs/PLA suture. However, in the later period, they degraded drastically and at last the mass had all disappeared. This was because at first the interior structure of PLA suture was dense, and the water molecule could hardly enter into PLA suture, so the degradation was very slow. However, with the time extension, the PLA suture was destroyed by degradation, and then some defects, such as pits, swellings and erosions holes (as shown in Figure 10), all emerged on the surface of the PLA suture. These provided the degradation solution and a chance to invade and destroy the PLA suture further. Thus the degradation ratio got more and more quickly. In a word, the sutures degraded lowly at early stage, and rapidly in the later period. Such characteristics of degradation just accorded with the requirements for suture. When the wound was healing and the suture was need to keep the wound tight at early stage, the suture degraded and lost their mass very slowly for making full use of their mechanical properties. In the later period, when the wound had healed up and the suture was no longer needed, the suture degraded and lost mass sharply, and then absorbed by the body at the right time.

When the mass loss ratio of sutures was 100% in Figure 10, the sutures degraded completely, and the total degradation periods of sutures, mixed with different contents of CNTs, were different, such as 49 weeks total degradation period for pure PLA suture, 63 weeks total degradation period for 0.5 wt% CNTs/PLA suture, 65 weeks total degradation period for 2 wt% CNTs/PLA suture and 73 weeks total degradation period for 1 wt% CNTs/PLA suture. It was shown that the total degradation period of CNTs/PLA sutures was longer than that of pure suture, due to involving CNTs, which indicated that the CNTs played a role in delaying degradation of PLA. This was because the added CNTs could improve the internal structure of PLA suture as shown in Section 3.1, such as increasing the crystallinity and orientation degree, refining and uniforming the grains and so on, which would slow down the degradation [34]. In addition, Figure 10 shows that the mass loss of 0.5 wt% and 2 wt% CNTs/PLA sutures was faster than that of 1 wt% CNTs/PLA suture, that is to say, the degradation rate of PLA suture containing 1 wt% CNTs was the slowest. This is because that the CNTs could improve the internal structure of PLA suture in the above analysis, and only moderate content (e.g., 1 wt%) of CNTs could improve the internal structure of PLA suture to a large extent, too much (e.g., 2 wt%) or too little (e.g., 0.5 wt%) content of CNTs could improve the internal structure of PLA suture just a little bit [46,47,48], therefore the 1 wt% CNTs/PLA suture degraded at the slowest rate and possessed the longest total degradation period.

### 3.6. The Chemical Structure of PLA Sutures during Degradation

**Figure 11:**
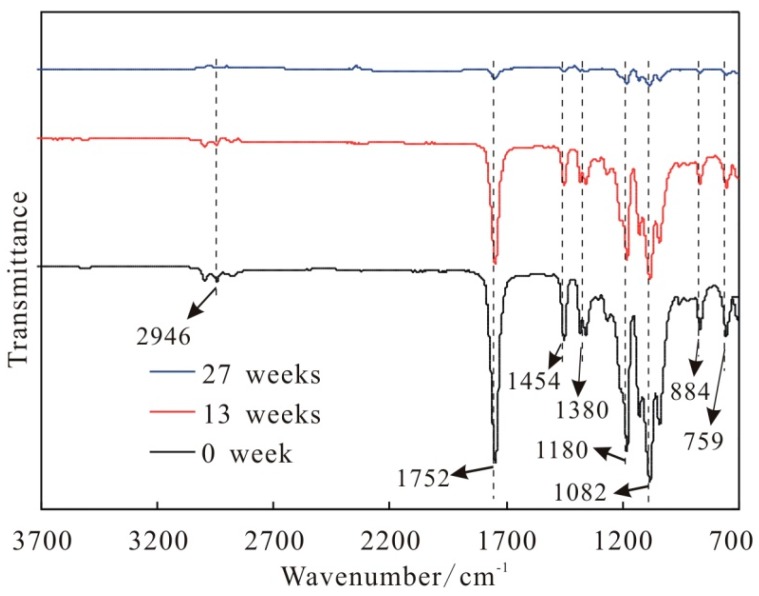
The fourier transform infrared spectra (FTIR) of 1 wt% CNTs/PLA sutures at different degradation time points.

In the process of degradation, the root cause of mass lose of suture is that the PLA molecules, as the main material of CNTs/PLA suture, were continuously degraded. The fourier transform infrared spectra (FTIR) of CNTs/PLA suture during degradation are shown in Figure 11.

Figure 11 shows that the three spectra in different degradation time (0, 13 and 27 weeks) presented the same characteristic absorption peaks of PLA molecule, including the characteristic absorption peak of “–C=O–” in ester group at 1752 cm^−1^, the stretching vibration absorption peak of “–C–O–” in ester group at 1181 cm^−1^ and 1082 cm^−1^, the deformation vibration absorption peak of “–CH_3_” at 1454 cm^−1^ and 1380 cm^−1^ and the deformation vibration absorption peak of “–CH_2_–” at 2946 cm^−1^. Furthermore, as time went on, these absorption peaks of PLA molecule were gradually weakened, which indicates that the PLA molecules were degraded gradually and the number of PLA molecules in suture decreased with time. According to relevant literatures [49,50,51], the PLA molecule could be hydrolyzed into lactic acid (LA), and the LA could be further metabolized by the body.

## 4. Conclusions

With the further degradation, the surface of pure PLA sutures appeared many pits and some severe swellings and corrosions. The added CNTs could slow down the structural damage on PLA suture during degradation. During the process of degradation, the surface of suture would form some structure defects, including peeling, swelling, corrosion hole and ditch. The PLA-base suture with moderate amounts (e.g., 1 wt%) of CNTs had the highest initial breaking strength. The breaking strength of all sutures decreased with the degradation time, and the pure PLA suture decreased obviously faster than CNTs/PLA sutures. The addition of CNTs into PLA matrix prolonged the degradation time of suture, and slowed down the degradation rate. The strength valid time of pure PLA suture was 13.5 weeks, and the time for the PLA sutures with CNTs was much longer. Especially for the PLA suture with 1 wt% CNTs, its valid time reached 26.6 weeks. The initial elongations (at degradation time of 0) of CNTs/PLA sutures were higher than that of pure PLA suture. With the degradation extension, the elongation of pure PLA suture declined obviously, and after 3 weeks its elongation had been very small (below 5%). The CNTs/PLA sutures’ elongation increased during the first 1–2 weeks, then decreased rapidly from the 3rd week, and at the 4th week the elongation had declined to 5% and even to 0% in later degradation time. The mass of pure PLA suture and CNTs/PLA suture lost slowly at early stage, but lost rapidly in the later period. When the mass loss ratio of sutures was 100%, the sutures degraded completely, and the total degradation periods of sutures, mixed with different contents of CNTs, were different, such as 49 weeks total degradation period for pure PLA suture, 63 weeks total degradation period for 0.5 wt% CNTs/PLA suture, 65 weeks total degradation period for 2 wt% CNTs/PLA suture and 73 weeks total degradation period for 1 wt% CNTs/PLA suture. The degradation rate (mass loss) of PLA suture containing 1 wt% CNTs was the slowest. During degradation, the PLA molecule would be degraded and hydrolyzed into lactic acid (LA). Finally, it was proved that CNTs could delay the degradation rate of suture and prolong the degradation period.

## Figures and Tables

**Table 1:** Healing time of different types of wounds.

Wound Type	Healing Time/Weeks
Appendsicitis	2–6
Hysteroma	8–11
Intestine	10–12
Intestinal polyp	12–15
Intervertebral disc herniation	12–16
Anorectal disease	15–18
Tendon	17–20
Cesarean	20–24
Osteorrhaphy	>40

**Table 2:** The spinning technical parameters.

Technical Parameters	Value
Zone 1 temperature/°C	180
Zone 2 temperature/°C	190
Zone 3 temperature/°C	192
Zone 4 temperature/°C	197
Elbow pipe temperature/°C	192
Spinning box temperature/°C	200
First hot roller temperature/°C	75
Second hot roller temperature/°C	80
Third hot roller temperature/°C	83
Annealing temperature/°C	65
Screw pressure/MPa	10.65
Melt temperature/°C	185
Screw frenquency/Hz	20.7
Metering pump frequency/Hz	60
First hot roller frequency/Hz	23.3
Second hot roller frequency/Hz	28
Third hot roller frequency/Hz	64
Winding frequency/Hz	100

**Table 3:** Fitting regression equations of breaking strength(y) and degradation time(x).

Items	Fitting Regression Equations	Degree of Fitting (R^2^)
Pure PLA suture	y = −0.9955x^2^ − 0.4807x + 389.2	0.985
0.5 wt% CNTs/PLA suture	y = −12.476x + 483.16	0.9755
1 wt% CNTs/PLA suture	y = −12.146x + 523.54	0.9899
2 wt% CNTs/PLA suture	y = −12.463x + 500.3	0.9855
